# Let’s Get Moving – The Effect of Sports and Exercise Therapy in the Treatment of Mental Illness on Physical Activity

**DOI:** 10.1192/j.eurpsy.2026.12011

**Published:** 2026-07-31

**Authors:** K. Friedrich, J. Krieger, V. Rößner-Ruff, C. Penkov, M. Wendt, M. Ziegenbein

**Affiliations:** 1 Research & Development; 2 Department of Sports and Physical Therapy, Wahrendorff Klinikum, Sehnde, Germany

## Abstract

**Introduction:**

Sports and Exercise Therapies (SET) have been indicated to have a therapeutic effect on the symptoms of mental illness, both as a complementary and as a stand-alone treatment. Furthermore, they have the capacity to exert a therapeutic or preventive effect in relation to (possible) physical comorbidities. Consequently, treatment guidelines recommend the integration of SET as a complementary therapy within a multimodal treatment approach. However, in Germany the data on the use of SET during treatment is inconsistent. It is therefore necessary to investigate the extent to which SET promotes physical activity in patients during (partial) inpatient treatment and, in particular, supports a physically active lifestyle beyond the treatment period, thus contributing to long-term stabilisation.

**Objectives:**

The present study investigates the extent to which SET influences patients’ physical activity during treatment. Moreover, the study examines the extent to which patients’ physical activity levels change before, during, and 12 weeks after the end of treatment.

**Methods:**

In a longitudinal study adults are surveyed using a self-reported questionnaire at the start of (partial) inpatient treatment, at the end of treatment, and 12 weeks after the end of treatment. The study is being conducted in a psychotherapeutic and psychosomatic specialist hospital in Lower Saxony, Germany. SET is a therapeutic intervention that is part of the treatment approach of the clinic. The level of physical activity is documented in minutes per week by means of a self-report questionnaire (BSA questionnaire). A distinction is made between everyday activities and sporting activities. Factors influencing physical activity are measured, including self-efficacy expectations, sport and exercise-related self-concordance, therapeutic alliance and the subjectively perceived effectiveness of SET.

**Results:**

The results of the statistical data analysis are presented.

**Conclusions:**

Based on the results, possible conclusions regarding the actual use of SET and its effects on physical activity are discussed. Implications for maintaining a physically active lifestyle beyond the end of treatment are considered, from a motivational psychology perspective.

**Disclosure of Interest:**

None Declared

